# Delayed diagnosis of brain tumor in a patient with flexor spasms and spastic foot drop

**DOI:** 10.4103/0972-2327.44564

**Published:** 2008

**Authors:** V. K. Paliwal, H. S. Malhotra, R. Sharma, Rakesh Shukla

**Affiliations:** Department of Neurology, Sanjay Gandhi Post Graduate Institute of Medical Sciences, Rae Bareli Road, Lucknow, Uttar Pradesh, India; 1 Department of Neurology, CSM Medical University, Formerly King George's Medical University, Lucknow, Uttar Pradesh, India

**Keywords:** Brain tumor, flexor spasms, spastic foot drop

## Abstract

Flexor spasms are involuntary muscle contractions comprising dorsiflexion at the ankle and flexion at the knee and the hip, occurring as a result of nociceptive spinal release reflex. The presence of flexor spasms generally suggests a lesion in the spinal cord. Foot drop is usually seen with lesions of lumbosacral roots, peripheral nerves or muscles. We hereby present a patient with a rare combination of spastic foot drop and flexor spasms due to a brain tumor. The possible underlying pathophysiological mechanisms resulting in flexor spasms due to a cerebral lesion are briefly discussed.

## Introduction

Flexor spasms are involuntary muscle contractions comprising dorsiflexion at the ankle and flexion at the knee and the hip, occurring as a result of nociceptive spinal release reflex.[1] Flexor spasms are generally seen in patients with spinal cord pathologies.[2] Rarely, they can be due to a cerebral lesion.[2] Foot drop is seen in patients with disc prolapse, neuropathies, spinal motor neuron disease and muscular dystrophies.[3] We report the simultaneous occurrence of flexor spasms and unilateral foot drop in a patient with brain tumor, which has not been reported before.

## Case Report

A thirty five-year-old gentleman presented to us with gradually progressive difficulty in walking for one and a half years, due to dragging of the right foot on the ground. This difficulty was associated with slippage of footwear from the right foot, with his being aware of it. Despite the weakness, the patient was able to walk with support and had no disability in terms of activities of daily living. On lying down, the patient had spontaneous involuntary painful drawing up of legs, in response to noxious or non-noxious stimuli. He noted these abnormal movements six months after the onset of the illness and since the last two months, these movements were frequent enough to disturb his sleep. The patient also had increased frequency, urgency and incontinence of urine for the past one year. There were no sensory complaints. There was no history of headache, vomiting, seizures, head or back trauma or fever. There was history of low backache for the last two years, which was intermittent, radiating along the back of right thigh, not increasing on straining or with change in posture and which used to respond to analgesics.

The patient was of average build, with normal results on general physical examination. Neurological examination revealed normal higher mental functions and cranial nerves. Mental status examination included attention span, memory, fund of acquired information, manipulation of old knowledge, social awareness and judgment, abstract thinking, praxis, right-left orientation and frontal lobe functions, all of which were found to be within normal limits. Fundus examination was normal. Motor system findings were localized to lower limbs. There was grade 3 spasticity (Ashworth scale) in the right lower limb and grade 2 spasticity in the left lower limb. There was MRC grade 0/5 power of dorsiflexors, evertors and invertors and 5/5 of plantiflexors at the right ankle, and normal power elsewhere. Flexor spasms were present in both the lower limbs (spasm score 4). Bilateral knee and ankle jerks were brisk. The right plantar response was extensor while the left plantar was not elicitable. Sensory system examination showed 1 cm difference in two point discrimination on the anterior aspect of the right leg, as compared to the corresponding points on the left side, with normal primary modalities of sensation on either side. No abnormality was detected in other cortical sensations such as stereognosis, graphesthesia, sensory attention and other gnostic or recognition functions. There was high steppage gait on the right side.

**Figure 1:**
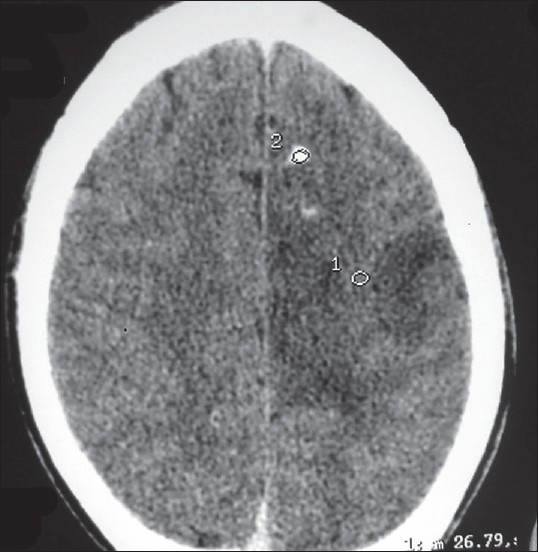
CECT brain showing large iso- to hypodense, poorly enhancing left fronto parietal, with areas of calcifications

**Figure 2:**
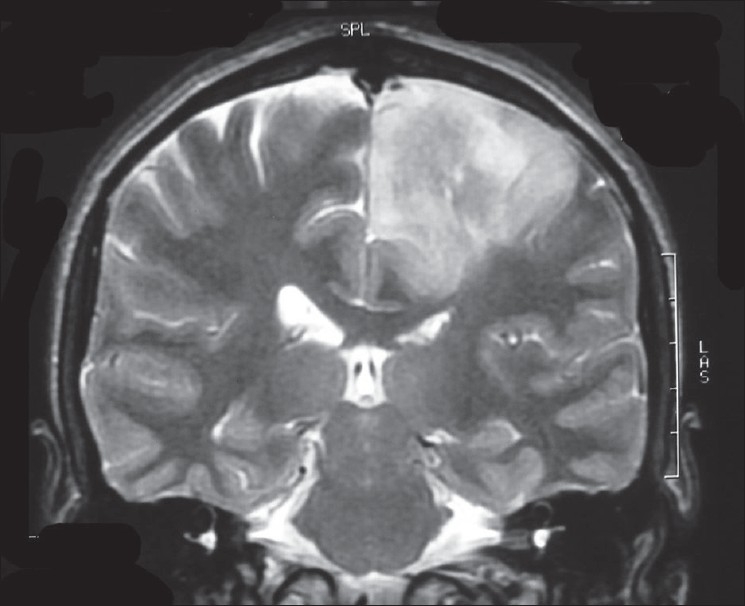
T2 weighted MRI brain coronal section showing parasagittal hyper intense mass with mass effect

Investigations revealed a normal hemogram and serum biochemistry. The patient had arrived with a diagnosis of noncompressive myelopathy, as the MRI scans of cervical and thoracic regions were normal. An MRI scan of the lumbosacral spine showed disc prolapse with minimal compression over thecal sac at L4-L5 level. The possibility of an intracranial lesion was considered due to abnormal cortical sensations in the presence of normal primary sensory modalities. A CT scan of the brain showed a large iso- to hypo-dense mass in the left fronto-parietal region, with areas of calcification [Figure 1]. Magnetic resonance imaging of the brain showed a heterogeneous space occupying lesion with areas of calcification, mass effect and minimal contrast enhancement [Figure 2]. Magnetic resonance spectroscopy demonstrated the elevation of the choline peak, with depression of N-acetyl aspartate (NAA) and creatine peaks. Directly observed EEG done on an analog machine at the time of occurrence of flexor spasms did not reveal any electrophysiological correlate. Biopsy of the mass was done through a burr hole in the left parietal bone over the mass, with a punch biopsy by a cruciate incision in the underlying dura. The biopsy revealed a grade II astrocytoma.

The patient was initially given intravenous dexamethasone and oral baclofen, with resultant reduction in spasticity and spasm score. Later, he received 60 gray of radiotherapy in 33 fractions. Chemotherapy with temozolomide was given along with radiotherapy. Follow-up examination at two months showed improvement in power at the right ankle joint, from grade 0 to grade II, and only occasional flexor spasms (spasm score 1).

## Discussion

Cerebral causes are known to cause foot drop,[3,4] but flexor spasms are thought entirely to be of spinal cord origin. This was the reason for the delayed diagnosis in our patient. The only suspicion of brain lesion was abnormal two point discrimination, involving the right leg.

Differential diagnoses of these involuntary movements are cortico-subcortical myoclonus and tonic motor spasms. Cortico-subcortical myoclonus is seen in a variety of disorders, occurring as a result of widespread encephalopathy and includes storage diseases, degenerative dementias, spinocerebellar degenerations, progressive myoclonic epilepsies, metabolic encephalopathies, toxic encephalopathies, infectious encephalopathies, posthypoxic encephalopathies etc. Tonic motor spasms have been described in patients of multiple sclerosis.[5,6] They are seen in limbs which do not show any upper motor neuron signs. The involuntary movements in our case were predictable, and inducible; they were not associated with features suggestive of an alternative diagnosis, were without any EEG correlate and they responded well to anti-spasticity drugs. The movements, therefore, appear most likely to be flexor spasms.

Flexor spasms (and extensor spasms) are painful, involuntary contractions of the muscles, which can be provoked by many causes like position, cutaneous stimuli, sleep onset, pain and infection.[2] Although spasms are most dramatic in patients with spinal cord injuries, these can occur with any type of upper motor neuron injury, including stroke and other forms of brain injury.[2] Pathophysiologically, flexor spasm is a flexor withdrawal reflex, a nociceptive spinal reflex, observed as on standing over a sharp object, comprising immediate dorsiflexion of the ankle and flexion at the knee and the hip, to withdraw from the stimulus. This spinal reflex is normally inhibited by various supra spinal fibers, dorsal reticulospinal pathway being the most important one. The motor areas of the cortex, through corticobulbar pathways, facilitate dorsal reticulospinal pathway, augmenting the net inhibitory drive down the spinal cord. Lesion of these corticobulbar pathways, either in the cortex or internal capsule, reduces the inhibitory drive and results in net excitation of spinal cord activity.[1]

Spasticity, deep tendon hyperreflexia and clonus are proprioceptive spinal reflexes, while flexor spasm is a normal nociceptive spinal reflex. Therefore, independent existence of these reflexes is possible. Flexor spasms usually accompany other features of the upper motor neuron involvement, like spasticity and hyperreflexia, because the main inhibitory pathway, i.e. dorsal reticulospinal tract, runs very close to the pyramidal tract in the spinal cord and the two are often involved together.

The lesion was present in the left parasagittal area, causing right sided spastic foot drop;[7,8] the extension of the lesion downwards, with mass effect and stretching of corpus callosum, may explain the occurrence of bilateral flexor spasms in our patient. Significant improvement in the spasm score, after treatment with radiotherapy and chemotherapy, further substantiates the cerebral origin of flexor spasm in the present case.

Thus, cortical lesions can rarely cause flexor spasms in conjunction with spasticity, weakness and other upper motor neuron features. Since flexor spasms are generally thought to occur in lesions of the spinal cord, a high index of suspicion should be kept in the case of those with a normal spinal MRI.
